# What non-clinical factors influence the general dentist–specialist relationship in Canada?

**DOI:** 10.1186/s12903-021-01782-y

**Published:** 2021-09-21

**Authors:** Harpinder Kaur, Sonica Singhal, Michael Glogauer, Amir Azarpazhooh, Carlos Quiñonez

**Affiliations:** 1 Dental Public Health, Faculty of Dentistry, University of Toronto, Toronto, ON Canada; 2 Public Health Ontario, Toronto, ON Canada; 3 Periodontics, Faculty of Dentistry, University of Toronto, Toronto, ON Canada

**Keywords:** General practice, Dental specialties, Professional practice, Interprofessional relations

## Abstract

**Background:**

The general dentist–specialist relationship is important for effective patient care and the professional environment. This study explores the non-clinical factors that may influence the general dentist–specialist relationship in Canada.

**Methods:**

A cross-sectional web-based survey of a sample of general dentists across Canada was conducted (N ≈ 11,300). The survey collected information on practitioner (e.g., age, gender, years of practice) and practice (e.g., location, ownership) factors. Two outcomes were assessed: not perceiving specialists as completely collegial and perceiving competitive pressure from specialists. Binary and multivariable logistic regression analysis was conducted.

**Results:**

A total of 1328 general dentists responded, yielding a response rate of 11.7%. The strongest associations for perceiving specialists as not completely collegial include being a practice owner (OR = 2.15, 95% CI 1.23, 3.74), working in two or more practices (OR = 1.69, 95% CI 1.07, 2.65), practicing in a small population center (OR = 0.46, 95% CI 0.22, 0.94), and contributing equally to the household income (OR = 0.47, 95% CI 0.26, 0.84). The strongest associations with perceiving medium/large competitive pressure from specialists include having a general practice residency or advanced education in general dentistry (OR = 2.00, 95% CI 1.17, 3.41) and having specialists in close proximity to the practice (OR = 2.52, 95% CI 1.12, 5.69).

**Conclusion:**

Practitioner and practice factors, mostly related to business and dental care market dynamics, are associated with the potential for strained relationships between general dentists and specialists in Canada. This study points to the need for dental professional organizations to openly discuss the current state of the dental care market, as it has important implications for the profession.

## Background

Collegial interprofessional relationships are important for delivering quality patient care and for the professional environment [1–3]. Such relationships are said to improve patient-centered care, the effectiveness of care, as well as the patient experiences and outcomes [1]. In dentistry, the main interface of the general dentist and specialist is through the referral process [2, 3]. During this interaction, both practitioners can exhibit positive or negative attitudes and behaviors toward one another. When each practitioner receives positive feedback from the other, their relationship can be considered favorable, resulting in mutual respect and confidence [2, 4, 5]. Negative feedback, on the contrary, can lead to strained feelings between practitioners.

Various factors might play a role in determining the nature of general dentist–specialist relationship. A general dentist’s perception of a specialist is known to be related to the specialist’s clinical skills, previous clinical success, style of communication, referred patients’ satisfaction, and the overall rapport with the specialist [6–9]. Professional competition may also impact a dentist’s relationship with specialists [10–12], while other factors like the availability of specialists, financial pressures, practice ownership, and practice location can also influence a practitioner’s clinical decisions [11–15].

Current trends within the Canadian and American dental care market appear to have the potential to negatively affect this interprofessional relationship [16]. Decreasing dentist-to-population ratios, the increasing costs of managing dental practices, changing professional demographics, and commercialism in dentistry are examples of such trends [16]. As a result, some dental professional organizations are suggesting that there are impacts on the general dentist–specialist relationship in the form of rising competition and diminished collegiality [16].

While previous studies [14, 15, 17–19] have explored various clinical and non-clinical factors that influence referrals and other clinical decisions, the dynamics of what influences the general dentist–specialist relationship remains unexplored. The aim of this study is thus to explore practitioner- and practice-related factors that may be associated with general dentists’ perceptions of their relationship with dental specialists in Canada.

## Methods

### Survey development and administration

This study comprises a web-based survey that collected data from general dentists in Canada. It was conducted through a partnership established between the Canadian Dental Specialties Association (CDSA), the Canadian Dental Association (CDA), and researchers (HK and CQ) at the Faculty of Dentistry, University of Toronto. The University of Toronto Research Ethics Board (REB) approved the research study (protocol #37318).

The survey was distributed in two waves in 2019 to all general dentists in the 2018 CDA register who had previously consented to receiving survey requests (N ≈ 11,300). An invitation email including a survey link was sent by the CDA, which directed dentists to the survey platform (ZohoSurvey®). Participation was completely voluntary, and the purpose and other details of the study were explained in the email. Clicking on the link and completing the survey was taken as informed consent. A reminder email was sent 2 weeks later.

The sample size was determined using the equation [20]: n = [(Np)(p)(1 − p)]/(Np − 1)(B/C)^2^ + (p)(1 − p)], where size of the population is represented by ‘Np’, the proportion of the population (50%) expected to choose one of two response categories is ‘p’, sampling error (3%) is ‘B’, and ‘z’ statistic (1.96) of the confidence interval the sample size is denoted by ‘C’. The sample size, n was estimated to be 976, assuming maximal variation and a standard confidence interval of 95 percent.

**Fig. 1:**
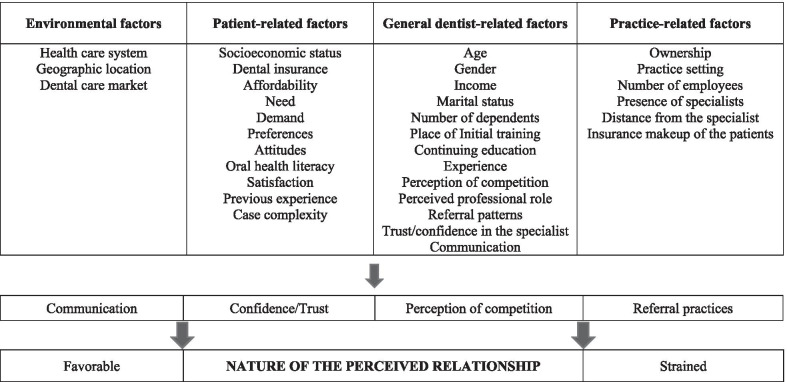
Conceptual framework of the factors involved in the general dentist–specialist relationship

A 47-item survey questionnaire was developed on the basis of a conceptual framework (Fig. 1) that, through a literature review, identified different factors (environmental, patient-, dentist-, and practice-related) that may affect, or are hypothesized to affect, the general dentist–specialist relationship [21]. In the absence of studies that explored this interprofessional relationship directly, we included factors shown in the literature to influence dentists’ referrals and clinical decisions [14, 15, 17–19]. The survey collected dentists’ socio-demographic information, professional characteristics, and their perceptions of confidence in, and competition with, specialists. The questionnaire was pilot-tested among a group of 12 general dentists and specialists to determine: (1) how long it took to complete, (2) if all questions were easy to understand, and (3) if any questions needed to be added. After minimal modifications, the questionnaire was translated into French and fielded by the CDA as discussed above.

### Outcome and exposure variables

We conceptualized the general dentist–specialist relationship by using four constructs: ‘communication’, ‘confidence/trust’, ‘perception of competition’ and ‘referrals’ [21]. This paper concentrates on the ‘perception of competition’ construct. It was assumed that the general dentist–specialist relationship was ‘strained’ when general dentists felt specialists to be competitive and not collegial towards them [21]. In this regard, two outcomes were assessed.

The first outcome concerned how general dentists perceived their relationship with specialists in the context of collegiality. The survey asked the question, “*Do you perceive specialists as colleagues or competitors?*” [14, 15]. A visual analogue scale (VAS) was used, where general dentists indicated their perceived relationship by marking an ‘X’ on a line ranging from 0 (completely collegial) to 100 (completely competitive) [14, 21]. During coding and analysis, the scale was converted into five categories: ‘completely collegial,’ ‘somewhat collegial,’ ‘neutral,’ ‘somewhat competitive,’ and ‘completely competitive’ [21]. Because the data were not normally distributed, the scale was dichotomized as ‘completely collegial’ (for those who marked between 0 and 24) and ‘competitive’ (for those who marked between 25 and 100), which differentiated between those who felt a strong level of collegiality in ideal terms and those who did not [21].

The second outcome concerned general dentists’ perceptions about the amount of competitive pressure they felt from specialists. General dentists were asked the question, “*In terms of competition, how much pressure do you feel from specialists?*” [15, 21]. The respondents were to select one of four categories: ‘no pressure,’ ‘small,’ ‘medium,’ and ‘large pressure’.

The exposure variables were categorical in nature and were grouped as practitioner (socio-demographic and professional) and practice characteristics (see Tables 1, 2 and 3).

**Table 1:** Descriptive characteristics of the survey sample

	Survey data (n, %)		CDA data (n, %)	
*Socio-demographic factors*				
Age	1123		19,992	
40 years and younger	278	24.8	6911	34.7
41–50	297	26.4	4969	24.9
51–60	316	28.1	4423	22.3
> 61	232	20.7	3609	18.1
Gender	1116		20,091	
Male	657	58.9	11,945	59.4
Female	459	41.1	8146	40.6
*Professional factors*				
Year of graduation	1281		19,927	
< 1996	673	52.5	8941	44.9
1997–2007	310	24.2	5083	25.5
2008–2018	298	23.3	5903	29.6
Place of initial training	1281		20,649	
Canadian dental faculty	951	74.2	15,413	74.6
American dental faculty	85	6.7	1784	8.6
International dental faculty	245	19.1	3452	16.7

**Table 2:** Binary and multivariable logistic regression presenting the odds of perceiving specialists as ‘not completely collegial’

	Binary		Multivariable	
	Odds ratio* (95% CI)	*p*	Odds ratio** (95% CI)	*p*
*Socio-demographic factors*				
Age				
40 years and younger (reference)	1.00		1.00	
41–50	1.67 (1.04, 2.69)	0.035	1.14 (0.67, 1.92)	0.628
51–60	1.25 (0.76, 2.05)	0.376	0.95 (0.54, 1.67)	0.849
> 61	0.87 (0.49, 1.55)	0.643	0.68 (0.35, 1.34)	0.265
Gender				
Male (reference)	1.00		1.00	
Female	0.65 (0.45, 0.94)	0.023	0.88 (0.57, 1.35)	0.551
Gross annual income				
< $149,000 (reference)	1.00		1.00	
$150,000–$249,000	0.75 (0.45, 1.24)	0.260	0.72 (0.42, 1.22)	0.220
> $250,000	1.91 (1.25, 2.93)	0.003	1.48 (0.91, 2.40)	0.112
Relationship status				
Single/non-married (reference)	1.00			
Married	0.93 (0.57, 1.52)	0.781		
Divorced/separated	1.37 (0.61, 3.04)	0.444		
Primary income-earner in the household				
Yes (reference)	1.00		1.00	
No	0.66 (0.37, 1.17)	0.151	0.84 (0.44, 1.60)	0.596
My partner and I contribute equally	0.42 (0.24, 0.73)	0.002	0.47 (0.26, 0.84)	0.011
Number of dependents				
0 (reference)	1.00			
1	0.61 (0.33, 1.13)	0.115		
2–4	1.44 (0.93, 2.23)	0.102		
5 or more	2.10 (0.91, 4.88)	0.083		
*Professional factors*				
Type of practitioner				
General dentist (reference)	1.00		1.00	
General dentist with GPR/AEGD	1.73 (1.06, 2.81)	0.027	1.63 (0.96, 2.75)	0.071
Place of initial training				
Canadian dental faculty (reference)	1.00			
American dental faculty	0.75 (0.35, 1.61)	0.454		
International dental faculty	1.10 (0.71, 1.71)	0.659		
Years of practice in Canada				
< 14 years (reference)	1.00			
15–29	1.01 (0.68, 1.51)	0.943		
> 30	0.71 (0.44, 1.13)	0.145		
Presence of student loans				
No, I do not have any student loans (reference)	1.00		1.00	
Yes	0.63 (0.44, 0.89)	0.010	0.58 (0.40, 0.85)	0.006
Hours of CE in a year				
Less than 30 (reference)	1.00			
More than 30	1.08 (0.71, 1.64)	0.724		
Involvement in a study club				
No (reference)	1.00			
Yes	1.37 (0.96, 1.94)	0.082		
Perception of pressure from other general dentists				
No pressure/small amount (reference)	1.00			
Medium/large pressure	1.14 (0.81, 1.62)	0.450		
*Practice factors*				
How many practices do you work in?				
1 (reference)	1.00		1.00	
2 or more	1.60 (1.08, 2.37)	0.018	1.69 (1.07, 2.65)	0.023
Perception about size of practice loans				
No outstanding practice loans (reference)	1.00		1.00	
Small	1.34 (0.77, 2.34)	0.301	0.90 (0.48, 1.68)	0.740
Medium	2.41 (1.49, 3.91)	< 0.001	1.46 (0.83, 2.56)	0.185
Large	2.16 (1.38, 3.38)	0.001	1.53 (0.86, 2.71)	0.147
Location of the primary practice				
Large population center (100,000 or greater) (reference)	1.00		1.00	
Medium population center (30,000–99,999)	1.25 (0.82, 1.91)	0.292	1.30 (0.83, 2.05)	0.250
Small population center (1000–29,999)	0.32 (0.17, 0.58)	< 0.001	0.46 (0.22, 0.94)	0.033
Practice ownership				
No, I do not own a practice (reference)	1.00		1.00	
Yes	2.63 (1.71, 4.04)	< 0.001	2.15 (1.23, 3.74)	0.007
Presence of specialists in close proximity (< 5 km) to the practice				
No (reference)	1.00		1.00	
Yes	2.85 (1.54, 5.28)	0.001	1.74 (0.83, 3.66)	0.143
Presence of in-house/visiting specialist				
No (reference)	1.00	0.955		
Yes	0.98 (0.62, 1.56)			

*Model 1 entered all the variables independently

**Model 2 entered significant variables from Model 1 (*p* < 0.05) as a block, adjusting for all variables simultaneously

**Table 3:** Binary and multivariable logistic regression presenting the odds of perceiving ‘medium/large competitive pressure’ from specialists

	Binary		Multivariable	
	Odds ratio* (95% CI)	*p*	Odds ratio** (95% CI)	*p*
*Socio-demographic factors*				
Age				
40 years and younger (reference)	1.00			
41–50	1.07 (0.65, 1.75)	0.801		
51–60	0.79 (0.47, 1.33)	0.378		
> 61	0.65 (0.36, 1.19)	0.163		
Gender				
Male (reference)	1.00			
Female	0.67 (0.45, 1.01)	0.053		
Gross annual income				
< $149,000 (reference)	1.00		1.00	
$150,000–$249,000	1.30 (0.77, 2.18)	0.331	1.40 (0.81, 2.41)	0.224
> $250,000	1.76 (1.08, 2.86)	0.024	1.68 (0.98, 2.88)	0.060
Relationship status				
Single/non-married (reference)	1.00			
Married	0.86 (0.51, 1.45)	0.570		
Divorced/separated	1.09 (0.45, 2.66)	0.844		
Primary income-earner in the household				
Yes (reference)	1.00			
No	0.61 (0.32, 1.19)	0.147		
My partner and I contribute equally	0.60 (0.35, 1.03)	0.064		
Number of dependents				
0 (reference)	1.00		1.00	
1	0.93 (0.48, 1.80)	0.819	0.84 (0.43, 1.66)	0.620
2–4	1.69 (1.02, 2.79)	0.042	1.39 (0.80, 2.40)	0.240
5 or more	3.21 (1.34, 7.69)	0.009	2.33 (0.91, 5.94)	0.077
*Professional factors*				
Type of practitioner				
General dentist (reference)	1.00		1.00	
General dentist with GPR/AEGD	2.04 (1.23, 3.38)	0.006	2.00 (1.17, 3.41)	0.011
Place of initial training				
Canadian dental faculty (reference)	1.00			
American dental faculty	0.69 (0.29, 1.65)	0.406		
International dental faculty	1.15 (0.72, 1.84)	0.570		
Years of practice in Canada				
< 14 years (reference)	1.00		1.00	
15–29	0.76 (0.49, 1.17)	0.209	0.63 (0.38, 1.04)	0.072
> 30	0.59 (0.35, 0.97)	0.039	0.71 (0.38, 1.31)	0.274
Presence of student loans				
No, I do not have any student loans (reference)	1.00			
Yes	1.03 (0.70, 1.52)	0.891		
Hours of CE in a year				
Less than 30 (reference)	1.00			
More than 30	0.71 (0.46, 1.09)	0.116		
Involvement in a study club				
No (reference)	1.00			
Yes	0.89 (0.60, 1.31)	0.554		
Perception of pressure from other general dentists				
No pressure/small amount (reference)	1.00			
Medium/large pressure	0.76 (0.52, 1.12)	0.170		
*Practice factors*				
How many practices do you work in?				
1 (reference)	1.00			
2 or more	1.52 (0.99, 2.33)	0.055		
Perception about size of practice loans				
No outstanding practice loans (reference)	1.00		1.00	
Small	0.93 (0.48, 1.80)	0.832	0.67 (0.33, 1.36)	0.268
Medium	1.45 (0.82, 2.57)	0.206	0.96 (0.50, 1.84)	0.902
Large	2.53 (1.60, 4.00)	< 0.001	1.79 (0.99, 3.24)	0.054
Location of the primary practice				
Large population center (100,000 or greater) (reference)	1.00		1.00	
Medium population center (30,000–99,999)	1.10 (0.69, 1.77)	0.692	1.03 (0.63, 1.69)	0.912
Small population center (1000–29,999)	0.44 (0.24, 0.80)	0.007	0.73 (0.36, 1.45)	0.366
Practice ownership				
No, I do not own a practice (reference)	1.00		1.00	
Yes	2.01 (1.29, 3.14)	0.002	1.73 (0.97, 3.08)	0.063
Presence of specialists in close proximity (< 5 km) to the practice				
No (reference)	1.00		1.00	
Yes	3.05 (1.51, 6.14)	0.002	2.52 (1.12, 5.69)	0.026
Presence of in-house/visiting specialist				
No (reference)	1.00			
Yes	1.11 (0.68, 1.81)	0.666		

*Model 1 entered all the variables independently

**Model 2 entered significant variables from Model 1 (*p* < 0.05) as a block, adjusting for all variables simultaneously

### Statistical analysis

Anonymized data was received from the CDA in Excel™ format. Data was analyzed using IBM® SPSS® Statistics. Spearman’s rho was used to explore the correlation between the exposure variables and outcomes. Next, logistic regression analyses (binary and multivariable) were used to determine the association of exposure variables with the odds of perceiving specialists as ‘not completely collegial’ and perceiving ‘medium/large competitive pressure’ from specialists. Binary logistic regression produced unadjusted odds ratios. Exposure variables with significant associations (*p* < 0.05) were then entered as a block into multivariable logistic regression to assess the dominant associations for both outcomes.

## Results

A total of 1328 surveys were received, yielding a response rate of 11.7%. After eliminating missing responses, 954 surveys were analyzed. To ensure the generalizability of the sample, we compared the demographic characteristics of the respondents with available census information on dentists from the CDA (Table 1). The survey sample was comparable in terms of gender and place of initial training but not with respect to age and year of graduation; the sample was skewed toward dentists who were 51–60 years of age versus 40 years and younger and those who graduated before 1996.

### General dentists, specialists, and collegiality

Table 2 presents the variables that were significantly associated with perceiving specialists as ‘not completely collegial’. At the binary level, the odds of perceiving specialists as not completely collegial increased among general dentists who were 41–50 years old, earned more than $250,000 per year, held a General Practice Residency (GPR) or Advanced Education in General Dentistry (AEGD), worked in two or more practices, perceived their practice loans to be either medium or large, owned a practice, and had specialists in close proximity to their practice. The odds decreased among female general dentists and those who contributed equally to their household income, had student loans, and practiced in less populated areas. In multivariable regression, the dominant factors associated with perceiving specialists as ‘not completely collegial’ include: working in two or more practices, which increased the odds by 69% (95% CI 1.07, 2.65); being a practice owner, which increased the odds by 115% (95% CI 1.23, 3.74); practicing in a small population center, which decreased the odds by 54% (95% CI 0.22, 0.94); contributing equally to the household income, which decreased the odds by 53% (95% CI 0.26, 0.84); and having a student loan, which decreased the odds by 42% (95% CI 0.40, 0.85).

### General dentists, specialists, and competition

Table 3 presents the variables that were significantly associated with perceiving ‘medium/large’ competitive pressure from specialists. The binary logistic regression revealed that the odds of perceiving medium/large competitive pressure from specialists increased among general dentists who earned more than $250,000 per year, had two or more dependents, held a GPR/AEGD, perceived their practice loans to be large, owned a practice, and had specialists in close proximity to their practice. The odds decreased among those who had more than 30 years of practice and were located in less populated areas. In multivariable regression, the dominant factors associated with perceiving medium/large competitive pressure from specialists include: having a GPR/AEGD, which increased the odds by 100% (95% CI 1.17, 3.41); and having specialists in close proximity to their practice, which increased the odds by 152% (95% CI 1.12, 5.69).

## Discussion

This study suggests the presence of associations between non-clinical factors and general dentists’ perceptions of dental specialists in Canada. Arguably, our findings can be explained by the financial pressures and implications of practicing dentistry, as well as the prevailing competition in the dental care market. For example, our results show that being a practice owner was associated with perceptions of both competition and collegiality with specialists. There is no doubt that owning a business usually predisposes one to diverse types of financial pressures [12, 15]. Research has shown that dentists who own a practice tend toward maximizing profit to assure the sustainability of their business [10, 13]. When financially challenged, it can thus be hypothesized that dentists might feel competitive pressure from specialists, view them less as colleagues and more as competitors, and thus resist referring to them. Some studies do suggest that the increasing costs of running or owning a dental practice contribute to a decline in the use of specialists [10, 16], and other evidence confirms that practice ownership can influence a dentist’s clinical decisions and treatment practices [11–13, 15]. Consequently, it would make sense that the perceptions of dentists toward specialists could be impacted by financial pressures and a competitive dental care market.

Certain situations might also drive a practitioner to react differently when facing professional and financial challenges. Our finding of decreased odds in perceiving specialists as not completely collegial among general dentists who contributed equally to their household finances compared to those who were primary income earners can be considered one possible scenario of financial challenges. Similarly, working in two or more practices was associated with increased odds of the same outcome, which can be observed as a professional challenge. Nevertheless, with a lack of corroborating evidence to support the above observations, we can only hypothesize that different situations may influence general dentists’ perceptions of their relationships with specialists.

Past studies have shown that the location of a dental practice predicts the rate of utilization of dental services [13, 22]. Practices in highly populated areas are more likely to provide larger numbers of services than those in less populated areas, possibly due to a competitive market [22, 23]. Our survey indicates that general dentists with practices located in small population centers did not perceive specialists as competitive or non-collegial. It can be assumed that areas with less population might indicate lower competition and a lesser availability of specialists and, therefore, more collegial environments. Likewise, general dentists in small population centers might have a stronger and established rapport with the limited number of specialists available.

Research has shown that a practitioner’s experience and advanced training impacts their referral patterns [24–27]. One systematic review reported a higher frequency of referrals from dentists who perceived that they had inadequate training compared to those who felt more confident in their skills [8]. Self-confidence is known to affect the interaction between general dentists and specialists as well [1, 4]. Thus, there is some logic to our finding that practicing with a GPR/AEGD is associated with perceiving specialists as competitive or non-collegial.

We also found that dentists who reported the presence of specialists in close proximity perceived medium/large competitive pressure from them. This is corroborated by the findings of previous studies [18, 19], which suggest a lower rate of referrals from practices closer to a specialist. Another possible explanation might be that practicing in the same area as specialists can burden general dentists in terms of maintaining patient volume, ultimately adding to the competitive pressure they perceive from specialists [18].

This study has several strengths and limitations. One strength was achieving the minimal sample size. While this was associated with a low response rate (11.7%), it is important to note that this response rate still compares well with those from web-based surveys among dental professionals [28–30]. Nevertheless, from the point of view of representativeness and generalizability, one cannot ignore the fact that the sample was biased toward older dentists and only included dentists who previously consented to receiving survey requests from the CDA. Our results were also dependent on self-reported data, increasing the potential for measurement error, such as social desirability and non-response bias. There was also no way to test for the latter. Further, our survey presents a one-sided assessment of the relationship between general dentists and specialists, and future work will require exploration of specialists’ perspectives. The generalizability of our findings to countries with different oral healthcare systems than that of Canada’s also poses as a limitation. We recognize that countries with comparable oral healthcare systems such as USA and Australia might present similar findings, yet the notion of a ‘strained’ relationship based on the perception of competition might not apply in countries with lesser competitive healthcare markets due their organization, financing, and/or delivery mechanisms. Finally, in the absence of any previous tools or constructs that explicitly assess the general dentist–specialist relationship, our suggested constructs to define the nature of this relationship present with issues of validity, but in their exploratory nature, also represent an opportunity to assess validity and develop instruments through future research. In this regard, qualitative research will be needed to develop the conceptual base of this area of inquiry in dentistry.

By identifying areas associated with a potentially strained relationship between general dentists and specialists, this study attempts to inform dental professional organizations. For instance, given the importance of interprofessional relationships and their potential impacts on patient care and on perceptions of dentistry’s professionalism, interventions to improve such relationships include: (1) educational (dental school curriculum can hone collaboration skills, stress the necessity of respecting one’s own professional limits), (2) regulatory (promoting regular communication between groups of practitioners, providing guidance on determining scopes of practice based on credentials and training, promoting healthy collaboration, assuring professional conduct), and (3) association-based (providing opportunities for continuing education (CE) and discussion to members on all of the above topics). Similarly, attention could be placed on helping current and future practitioners navigate through the financial and professional challenges they can face, given the influence of the issues highlighted in this paper.

## Conclusions

It appears that practitioner and practice factors related to the business of dentistry and dental care market dynamics may be involved in negatively influencing the relationship between general dentists and dental specialists in Canada.

## Data Availability

All relevant data/information has been included in this manuscript. However, the dataset used and/or analysed during this study are available from the corresponding author on reasonable request.

## Abbreviations

AEGD: Advanced education in general dentistry
CDA: Canadian dental association
CDSA: Canadian dental specialties association
GPR: General practice residency
VAS: Visual analogue scale
